# Treatment of Leucorrhœa

**Published:** 1846-10

**Authors:** 


### FACTS FROM CORRESPONDENTS.

### TREATMENT OF LEUCORRHŒA.

Dr. Osborne, of Erie, Alabama, has found the following treatment of *Leucorrhœa* uniformly successful:

To 2 ounces of a decoction of red oak bark add 10 drops of the tincture of muriate of iron, and inject into the vagina two or three times daily. I am apprized of the incompatibility of the two articles, but my experience is that they form a compound which acts like a specific in this troublesome affection. I cannot help regarding the remedy as one entitled to the confidence of the profession.

Erie, Ala., August, 1846.

